# Biomarkers of Cardiac Metabolic Flexibility in Health, HFrEF and HFpEF

**DOI:** 10.3390/ijms27020879

**Published:** 2026-01-15

**Authors:** Hyeong Rok Yun, Manish Kumar Singh, Sunhee Han, Jyotsna S. Ranbhise, Joohun Ha, Sung Soo Kim, Insug Kang

**Affiliations:** 1 Department of Biochemistry and Molecular Biology, School of Medicine, Kyung Hee University, Seoul 02447, Republic of Korea; foryou018@naver.com (H.R.Y.);; 2 Biomedical Science Institute, Kyung Hee University, Seoul 02447, Republic of Korea; 3 Department of Biomedical Science, Graduate School, Kyung Hee University, Seoul 02447, Republic of Korea

**Keywords:** metabolic flexibility, heart failure, precision medicine, dynamic profiling

## Abstract

Cardiac metabolic flexibility is a key determinant of myocardial energetic resilience. In heart failure with reduced ejection fraction (HFrEF), intrinsic mitochondrial dysfunction and lipotoxicity compromise oxidative capacity. In contrast, heart failure with preserved ejection fraction (HFpEF) is orchestrated primarily by systemic comorbidities and coronary microvascular dysfunction, which decouple glycolysis from glucose oxidation. This review integrates these distinct pathophysiologies into a comprehensive biomarker framework. Beyond core hemodynamic markers, we detail indices of metabolic flux (ketones, acylcarnitines, branched-chain amino acids), endothelial injury, and fibrosis. We further prose a shift from static, isolated measurements to dynamic functional profiling using standardized challenges (e.g., mixed-meal or exercise tests) to quantify metabolic suppression and recovery kinetics. This structured hierarchy enables phenotype-tailored risk stratification and guides mechanism-based precision therapies in the era of personalized medicine.

## 1. Introduction

The heart is one of the most metabolically active organs in the body and continuously synthesizes substantial quantities of adenosine triphosphate (ATP) to sustain rhythmic cycles of contraction and relaxation [1]. Myocardial fuel utilization is primarily determined by metabolic flexibility, defined as the capacity to rapidly reallocate fuel use in response to changes in energetic demand, substrate availability, oxygen supply and hormonal or environmental cues, rather than reliance on single dominant pathway [2,3]. Under normal conditions, myocardial ATP production is dominated by fatty acid oxidation. However, elevations in insulin after feeding, hemodynamic loading that increases oxygen demand or limited oxygen delivery induce a shift in substrate utilization from fatty acids to glucose and lactate [4,5]. Oxidation of ketone bodies, primarily β-hydroxybutyrate (BHB) and acetoacetate, provides a rapidly accessible auxiliary substrate, and the dynamics of substrate switching are tightly coupled to microvascular function, mitochondrial capacity and coupling efficiency, intracellular redox balance and autonomic and endocrine signaling [6,7].

A decline in metabolic flexibility entrains the myocardium to a rigid pattern of substrate utilization, thereby precipitating a critical discordance between ATP supply and demand [8]. This metabolic rigidity not only fosters the lipotoxic accumulation of diacylglycerols and ceramides but also amplifies mitochondrial reactive oxygen species (mtROS) production. Consequently, it triggers microvascular inflammation with endothelial dysfunction and drives progressive interstitial fibrosis, ultimately converging on heart failure (HF) phenotypes characterized by both diastolic and systolic dysfunction [9,10,11]. HFrEF and HFpEF are both characterized by a common energy supply–demand mismatch, while differing in predominant metabolic defects and in the principal pathophysiologic pathways that drive disease progression [12,13].

Biomarkers provide noninvasive insight into the complex remodeling of cardiac metabolism and structure. Clinically meaningful information reflects the pattern of changes across metabolic and pathophysiologic domains, rather than the absolute value of an isolated marker, and can be used to refine diagnosis, risk stratification, monitoring of treatment response, and phenotypic clustering [14,15].

In this review, we aim to integrate cardiac metabolic flexibility across health, HFrEF, and HFpEF, and relate it to mechanism-based biomarker categories, with the goal of providing a structure that can be applied to clinical decision-making.

## 2. Cardiac Metabolism

In the normal heart, mitochondrial β-oxidation of long-chain fatty acids (LCFAs) supplies most of the ATP, while the capacity to shift oxidative flux toward glucose, lactate, and ketone bodies is preserved and can be rapidly mobilized in response to changing energetic demands [16,17,18]. The myocardium is highly specialized for oxidative phosphorylation, such that mitochondria occupy a large proportion of cardiomyocyte volume, and approximately 95% of myocardial oxygen consumption is devoted to ATP production [19,20]. LCFAs reach the myocardium as albumin-bound non-esterified fatty acids or as lipolytic products of triglyceride-rich lipoproteins, including chylomicrons and very-low-density lipoproteins (VLDL) [21]. Upon arrival in the myocardial interstitium, LCFAs are transported into cardiomyocytes primarily via cluster of differentiation 36 (CD36) and are subsequently sequestered and trafficked within the cytosol by fatty acid-binding proteins (FABPs) [22]. At the outer mitochondrial membrane, long-chain acyl-CoA synthetases (ACSLs) catalyze the formation of acyl-CoA esters from LCFAs, and the resulting acyl-CoA esters are ten translocated into the mitochondrial matrix via the carnitine shuttle, which comprises carnitine palmitoyltransferase-1 (CPT1), the carnitine–acylcarnitine translocase (CACT) and carnitine palmitoyltransferase-2 (CPT2) [23,24]. Within the mitochondrial matrix, long-chain acyl-CoA esters undergo repeated cycles of β-oxidation, generating acetyl-CoA, NADH and FADH_2_. Acetyl-CoA subsequently enters the tricarboxylic acid (TCA) cycle, whereas NADH and FADH_2_ donate electrons to the mitochondrial electron transport chain (mETC), processes that collectively underwrite the majority of myocardial ATP production [13,24,25]. However, despite its high ATP yield per mole of substrate, fatty acid oxidation is relatively oxygen-intensive. Accordingly, under conditions of hypoxia, acute hemodynamic stress, or abrupt increases in workload, glucose oxidation and lactate utilization are rapidly promoted [18,26,27,28,29]. Glucose oxidation is regulated by glucose transporter type 1 and 4 (GLUT1 and GLUT4)-mediated uptake, glycolytic flux and reversible phosphorylation of pyruvate dehydrogenase (PDH) complex, which is inhibited by PDH kinases (PDKs) and reactivated by PDH phosphatases [30,31,32,33]. During exercise, lactate generated by skeletal muscle is rapidly taken up via monocarboxylate transporter 1 and 4 (MCT1/4) and serves as an immediately oxidizable fuel. During fasting or carbohydrate restriction, ketone bodies are recruited as a rapidly available auxiliary substrate and are oxidized via BHB dehydrogenase 1 (BDH1)–succinyl-CoA:3-ketoacid CoA transferase (SCOT) pathway [34,35,36].

Cardiac substrate switching is orchestrated through a hierarchical network involving substrate competition, energy sensing and mitochondrial fine-tuning. Key regulatory mechanisms and signaling pathways governing these substrate shifts are summarized in Table 1. At the level of substrate entry, the Randle cycle and the Malonyl-CoA/CPT axis determine the competitive balance between glucose and fatty acid uptake. Upstream of these checkpoints, adenosine monophosphate-activated kinase (AMPK) and mechanistic target of rapamycin complex 1 (mTORC1) function as central energy sensors that modulate metabolic flux in response to nutrient availability. Within the mitochondria, flux is further regulated by Ca^2+^-sensitive TCA dehydrogenases [PDH, isocitrate dehydrogenase (IDH) and α-ketoglutarate dehydrogenase (α-KGDH)] and post-translational modifications (Table 1). Specifically, the mitochondrial acylation-sirtuin axis, including SIRT3 and SIRT5, dynamically recalibrates enzymatic activity through deacetylation and desuccinylation, thereby ensuring coupling efficiency and redox balance [13,37,38,39,40]. In HF, disruption of the malonyl-CoA-CPT1 axis, together with chronic upregulation of PDH kinase 4 (PDK4), has been consistently linked to an “inefficient substrate predominance” phenotype, in which reliance on fatty acid oxidation is excessive and glucose oxidation is concurrently suppressed [41,42]. Acylation-based post-translational modifications (PTM) of mitochondrial proteins, including acetylation, succinylation, and β-hydroxybutyrylation, modulate the efficiency of the TCA cycle and mETC, with deacylases such as SIRT3 and SIRT5 serving as key regulators of these processes [43,44,45,46,47,48]. Proteomic studies in cardiac disease models indicate that SIRT3- and SIRT5-dependent remodeling of lysine acetylation and succinylation dynamically recalibrates TCA dehydrogenase and respiratory chain complex activities. These modifications also modulate reactive oxygen species (ROS) production and regulate stress-adaptation mechanisms [49,50,51,52,53]. Collectively, these findings implicate the mitochondrial acylation PTM–SIRT axis as a central fine-tuner of cardiac metabolic flexibility. Mitochondrial mass and quality are sustained by dynamic balance between biogenesis driven by the PGC-1α/nuclear factor erythroid-2-related factors (NRFs)/mitochondrial transcription factor A (TFAM) pathway and PTEN-induced putative kinase 1 (PINK1)/parkin RBR ubiquitin protein ligase (Parkin)-mediated mitophagy [54,55,56,57]. Disruption of this balance promotes excessive ROS production, destabilization of the mitochondrial membrane potential and increased variability in ATP generation, ultimately diminishing metabolic flexibility [58,59,60]. Parkin-independent quality control pathways have also been delineated, and the relative contribution of individual mechanisms likely vary across physiological and pathological states [61,62]. Cardiac energetics is tightly integrated with excitation–contraction coupling. ATP hydrolysis by sarcoplasmic/endoplasmic reticulum Ca^2+^-ATPase 2a (SERCA2a) and the Na^+^/K^+^-ATPase is required to maintain the cardiac action potential and Ca^2+^ cycling [63,64,65]. The microvascular–endothelial nitric oxide (NO)/cyclic guanosine monophosphate (cGMP)/protein kinase G (PKG) signaling maintains myocardial oxygen and substrate delivery, thereby preserving titin compliance. Disruption of NO/cGMP/PKG signaling during systemic metabolic dysfunction is regarded as a major driver of diastolic dysfunction [10,66,67,68].

Metabolic dysfunction most commonly arises from an imbalance between substrate supply and disposal [69,70]. In obesity and insulin resistance, fatty acid influx chronically exceeds mitochondrial oxidative capacity and coupling efficiency, leading to the accumulation of bioactive lipids such as long-chain acylcarnitines (LCACs), diacylglycerols and ceramides, which disrupt membrane-associated signaling cascades and perturb intracellular Ca^2+^ homeostasis [69,71,72,73]. In parallel, the redox balance of the NADH/NAD^+^ and NADPH/NADP^+^ couples is progressively constrained, the PDH complex is maintained in an inactive state, and lactate accumulates, thereby limiting the capacity to upregulate myocardial carbohydrate oxidation as energetic demand increases [74,75,76,77]. Endothelial dysfunction, oxidative stress and glucolipotoxicity promote coronary microvascular inflammation and fibrosis, thereby elevating filling pressures and contributing to diastolic dysfunction [9,78]. Epicardial adipose tissue and mediators arising from the gut–liver axis, including inflammatory cytokines, adipokines and gut-derived metabolites such as trimethylamine N-oxide (TMAO), are increasingly recognized as contributors to coronary microvascular dysfunction [79,80,81]. Aging and sex further modulate mitochondrial biogenesis, capillary density and the responsiveness of energy-sensing networks, thereby diminishing the speed and reversibility of substrate switching [5,82,83]. Taken together, crosstalk among the liver, adipose tissue and the gut regulates substrate accessibility and the balance of substrate oxidation, underscoring that cardiac metabolism must be interpreted within the integrated framework of whole-body metabolic homeostasis.

**Figure 1:**
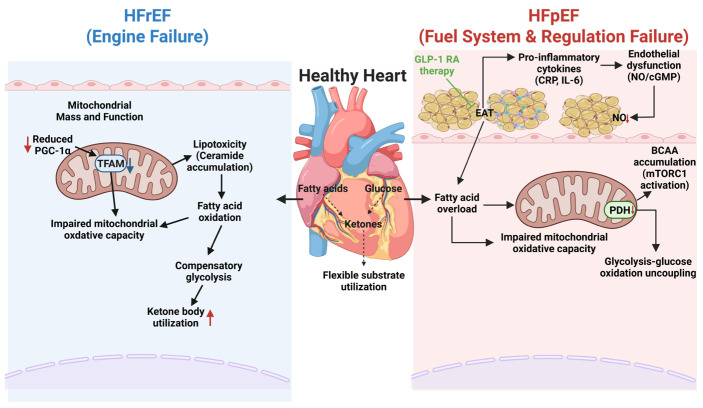
Divergent pathophysiological landscapes of metabolic inflexibility in heart failure with reduced ejection fraction (HFrEF) versus heart failure with preserved ejection fraction (HFpEF). The schematic delineates the distinct etiologies of metabolic dysfunction that drive heart failure phenotypes compared to the flexible substrate utilization in a healthy heart (center). Left Panel (HFrEF): The “Engine Failure” phenotype is characterized by intrinsic mitochondrial dysfunction, including mitochondrial rarefaction and downregulated biogenesis mediated by the peroxisome proliferator-activated receptor-gamma coactivator 1-alpha (PGC-1α) and mitochondrial transcription factor A (TFAM) axis. This bioenergetic deficit precipitates lipotoxic accumulation of ceramides and a compensatory, yet insufficient, shift towards glycolysis and ketone body oxidation to sustain contractile function. Right Panel (HFpEF): The “Fuel System and Regulation Failure” phenotype is driven by systemic metabolic comorbidities. Expansion and inflammation of epicardial adipose tissue (EAT) result in the paracrine secretion of pro-inflammatory cytokines, specifically interleukin-6 (IL-6) and tumor necrosis factor-alpha (TNF-α), which induce coronary microvascular endothelial dysfunction characterized by impaired nitric oxide (NO) and cyclic guanosine monophosphate (cGMP) signaling. At the cardiomyocyte level, this leads to fatty acid overload, accumulation of branched-chain amino acids (BCAAs) via activation of mechanistic target of rapamycin complex 1 (mTORC1), and the uncoupling of glycolysis from glucose oxidation due to inhibition of the pyruvate dehydrogenase (PDH) complex. The green arrow highlights the therapeutic mechanism of glucagon-like peptide-1 receptor agonists (GLP-1 RAs), which mitigate this cascade by reducing EAT volume and suppressing local inflammation, thereby restoring metabolic homeostasis. (Figure created in Biorender. Hyeong Rok Yun. (2025) https://app.biorender.com/illustrations/canvas-beta/69316def39f1cfda74bfb746, accessed on 13 January 2026).

Overall, the heart operates as a hybrid metabolic engine with default reliance on fatty acid oxidation, while glucose, lactate and ketone bodies can be rapidly recruited as auxiliary substrates. The metabolic flexibility system is sustained by coordinated regulatory networks, adequate mitochondrial mass and functional integrity and preserved microvascular-endothelial function. An overview of these metabolic mechanisms and their dysregulation in heart failure is presented in Figure 1.

## 3. HFpEF and HFrEF

### 3.1. Heart Failure with Reduced Ejection Fraction

HFrEF is defined by an left ventricular ejection fraction (LVEF) ≤ 40% and reflects impaired systolic reserve, leading to inadequate cardiac output relative to systemic demand [84]. In HFrEF, a pathophysiologic hallmark is a concomitant reduction in mitochondrial mass, functional integrity and reserve capacity, which limits myocardial ATP production even in the presence of adequate oxygen delivery [85,86,87]. Downregulation of the PGC-1α/NRF/TFAM transcriptional axis, together with dysregulated mitophagy, diminishes the pool of functional mitochondria, impairs mETC coupling efficiency and reduces high-energy phosphate reserve, as evidenced by a decline in the phosphocreatine (PCr)-to-ATP ratio (PCr/ATP) [88,89,90]. The mismatch between sustained fatty acid uptake and diminished oxidative capacity leads to the accumulation of toxic lipid intermediates, including ceramides and diacylglycerols. This phenomenon, termed cardiac lipotoxicity, directly induces apoptosis of cardiomyocytes, impairs insulin signaling, and exacerbates contractile dysfunction [73,91,92]. Notably, substrate utilization exhibits dynamic temporal evolution. While reliance on fatty acid oxidation is sustained in early stages, thereby exacerbating lipotoxic injury, progressive mitochondrial respiratory failure in advanced HF ultimately precipitates a decline in oxidative capacity and a compensatory reversion to glycolysis [3,93]. However, compensatory changes remain insufficient to fully support systolic energy demand. In parallel, abnormalities in sarcoplasmic reticulum (SR)–mitochondria coupling and persistent Ca^2+^ leak increase the energetic demand of excitation–contraction coupling, while ventricular dilation and elevated wall stress lead to additional increases in myocardial oxygen consumption [94,95,96,97,98,99]. Coronary microvascular dysfunction and oxidative stress exacerbate perfusion–metabolism mismatch, whereas sustained activation of the sympathetic nervous system together with the renin–angiotensin–aldosterone system (RAAS) promotes progression from an initially compensatory response to a self-perpetuating maladaptive state [100,101]. A persistent imbalance between energy supply and demand perpetuates the structural and electrical instability that characterizes HFrEF [102,103,104]. Beyond glucose and fatty acids, defects in branched-chain amino acid (BCAA) catabolism have emerged as a significant metabolic signature of HFrEF. Downregulation of mitochondrial BCAA clearance enzymes leads to the accumulation of BCAAs and branched-chain α -keto acids (BCKAs), which suppresses glucose oxidation and promotes oxidative stress via mTORC1 activation [105,106,107,108,109]. Under these energy-deficient conditions, HFrEF increases reliance on ketone bodies as oxidative substrates, a phenomenon that has attracted substantial interest, yet remains debated. Some studies have demonstrated that acute BHB infusion produces transient improvements in cardiac output and hemodynamic parameters, suggesting that ketone bodies may serve as an adaptive auxiliary fuel [6,110,111,112]. However, the role of ketone metabolism in HFrEF remains a subject of active debate. While acute hemodynamic benefits are evident, it is unclear whether the increased ketone utilization is predominantly adaptive or instead marks advanced disease severity and systemic metabolic stress. Furthermore, the long-term efficacy of ketone supplementation on ventricular remodeling and clinical outcomes has not yet been established in large-scale randomized trials.

### 3.2. Heart Failure with Preserved Ejection Fraction

HFpEF is a clinical syndrome characterized by typical symptoms and signs of heart failure despite a preserved LVEF ≥ 50%, accompanied by elevated left ventricular filling pressure that reflects underlying diastolic dysfunction [84]. HFpEF is closely linked to systemic cardiometabolic disease, particularly obesity, type 2 diabetes, hypertension, aging, and chronic kidney disease [113,114,115]. The HFpEF phenotype is predominantly governed by comorbidity-associated chronic systemic inflammation, endothelial activation and coronary microvascular dysfunction, which collectively reduce NO and attenuate signaling downstream of cGMP/PKG within cardiomyocytes [10,116,117]. Recent single-cell analyses have demonstrated that epicardial adipose tissue (EAT) undergoes expansion and phenotypic remodeling, becoming a local source of pro-inflammatory cytokines that drive the recruitment of pro-inflammatory and profibrotic macrophage populations and thereby aggravate coronary microvascular injury [118,119,120,121]. In this context, glucagon-like peptide-1 receptor agonists (GLP-1 RAs) have emerged as a targeted intervention for the obesity-driven HFpEF phenotype [122,123]. Beyond systemic weight loss, GLP-1 RAs exert direct pleiotropic effects on the cardiometabolic axis, including the reduction in epicardial adipose tissue volume and the suppression of systemic inflammatory cytokines (e.g., CRP), thereby mitigating the microvascular endothelial activation. Metabolically, GLP-1 signaling enhances myocardial insulin sensitivity and promotes glucose uptake via AMPK activation, potentially restoring the disrupted coupling between glycolysis and glucose oxidation and re-establishing metabolic flexibility against lipotoxic stress [124,125]. Hypophosphorylation of titin, together with altered post-translational modification of sarcomeric and cytoskeletal proteins, increases passive myofilament stiffness, whereas interstitial fibrosis and perivascular collagen deposition increase diastolic LV stiffness and impair ventricular-arterial coupling. Thus, in HFpEF, elevated wall stress, impaired relaxation and concentric remodeling chronically increase myocardial energy demand during exercise, while ejection fraction may remain preserved at rest [126,127,128]. Metabolically, HFpEF is characterized by a pronounced loss of flexibility, with substrate utilization closely determined by the underlying comorbidities. In obesity- and diabetes-related HFpEF, increased flux of non-esterified fatty acids from adipose tissue, together with increased CD36-mediated fatty acid uptake at the sarcolemma, exceeds mitochondrial oxidative capacity [129,130,131]. An imbalance between fatty acid delivery and oxidative capacity leads to accumulation of LCACs, diacylglycerols and ceramides. These metabolites disrupt membrane signaling microdomains, perturb Ca^2+^ handling and induce oxidative stress and chronic inflammation. This cascade creates a pattern of myocardial and systemic abnormalities tightly linked to the HFpEF phenotype, which is notably less prominent in HFrEF [71,72,132,133]. In multiple preclinical models and human HFpEF, fatty acid and glucose oxidation become dissociated from increased glycolytic flux, with glycolysis being upregulated while pyruvate entry into the TCA cycle is limited by PDK-mediated inhibition of PDH and insulin resistance. Such metabolic uncoupling reduces the efficiency of ATP production per molecule of oxygen consumed and promotes lactate accumulation and intracellular acidosis during exertion [134,135,136]. Energetically, HFpEF hearts exhibit a reduction in myocardial PCr/ATP similar in magnitude to that in HFrEF, indicating a global impairment of high-energy phosphate reserves even at rest [137,138]. Notably, patients with diabetes and concentric left ventricular remodeling, even in the absence of overt HFpEF, already exhibit reduced PCr/ATP ratios, suggesting that energetic deficit arises early along the HFpEF continuum and does not simply reflect advanced hemodynamic decompensation [12,139,140,141]. Emerging evidence also implicates defective branched-chain amino acid (BCAA) catabolism as a metabolic hallmark of HFpEF, with increased myocardial BCAA levels and reduced downstream mitochondrial acylcarnitine catabolites, consistent with impaired mitochondrial BCAA oxidation. In both clinical and experimental HFpEF, reduced activity of BCAA-catabolizing enzymes and dysregulated BCKA dehydrogenase (BCKDH) have been linked to activation of mTORC1, increased oxidative stress and adverse remodeling. Consistent with these findings, impaired BCAA catabolism in lymphatic endothelial cells promotes lymphatic dysfunction and HFpEF-like features in preclinical models [12,142,143,144]. In HFpEF, as in HFrEF, increased reliance on ketone metabolism likely reflects a secondary adaptation to energetic deficit rather than a primary driver of disease. Levels of peripheral ketone bodies are often increased, and experimental studies indicate that ketone bodies can partially support myocardial ATP production under conditions of reduced glucose and fatty acid oxidation. However, in HFpEF, oxidation of ketone bodies contributes only minimally to myocardial ATP production, and energetic efficiency remains impaired under conditions of persistent substrate mismatch and microvascular dysfunction [7,145,146,147].

## 4. Biomarker

### 4.1. Core Clinical Biomarkers

Natriuretic peptides, specifically B-type natriuretic peptide (BNP) and N-terminal pro-B-type natriuretic peptide (NT-proBNP), are widely regarded as the gold-standard biomarkers of myocardial wall stress and ventricular filling pressures [148]. Values are often disproportionately low in obesity, reflecting physiological suppression, whereas advanced age, chronic kidney disease (CKD), and atrial fibrillation (AF) are typically associated with higher concentrations. As a result, reliance on a single absolute cutoff lacks precise clinical discrimination power [149,150,151,152]. Similarly, high-sensitivity cardiac troponin (hs-cTn) assays detect subclinical myocardial injury related to microvascular ischemia and wall stress [153,154,155]. Within the cardio-renal axis, cystatin C (Cys-C) offers superior utility as a filtration marker less influenced by sarcopenia or muscle mass [156,157]. The urinary albumin-to-creatinine ratio (uACR) is a useful biomarker of systemic microvascular leak and congestion and is closely associated with long-term prognosis [158,159]. Emerging congestion-related biomarkers, including mid-regional pro-adrenomedullin (MR-proADM) and carbohydrate antigen 125 (CA125), reflect venous and lymphatic congestion as well as interstitial edema [160,161,162]. Collectively, this core panel establishes the hemodynamic baseline necessary for the accurate interpretation of metabolic shifts.

### 4.2. Biomarkers of Metabolic Flexibility and the Endothelial-Microvascular Axis

Metabolic flexibility is inadequately characterized by static fasting measurements alone [163,164]. Circulating concentrations of ketone bodies provide an indirect index of the mitochondrial NADH/NAD^+^ redox state [165,166]. However, the diagnostic utility is maximized not by fasting levels but by the suppression kinetics post-challenge. A blunted suppression of ketogenesis after a meal signifies metabolic inflexibility, which is a hallmark of insulin-resistant HFpEF. Direct assessment of metabolic flexibility relies on analyzing suppression and recovery trajectories after a standardized physiological challenge, such as a mixed meal or brief bouts of light exercise. Lactate and the lactate-to-pyruvate ratio provide information on PDH activity and cytosolic redox state, but are highly sensitive to recent physical activity, emotional stress, and venous congestion, making rigorous standardization of pre-analytical conditions essential [164,167]. Persistent elevation of LCACs (e.g., C16, C18:1) indicates a mismatch between fatty acid delivery and mitochondrial β-oxidation capacity and has been repeatedly observed in obesity, diabetes, and HFpEF [168,169,170]. However, the interpretation of single-point absolute values is limited by inter-platform variability and dietary factors. In addition, profiles of BCAAs and BCKAs, together with short-chain acylcarnitines such as C3 (propionylcarnitine) and C5 (isovalerylcarnitine), allow detailed characterization of BCAA oxidative flux and insulin-resistant metabolic phenotypes. Mechanistically, while elevated LCACs reflect a bottleneck in fatty acid β-oxidation, BCAA accumulation specifically points to defective catabolic enzyme activity that promotes oxidative stress via mTORC1 activation. 2-Hydroxybutyrate (α-hydroxybutyrate) is an early marker of redox stress and insulin resistance that reflects compensation within the glutathione pathway and increased NADPH demand. The dynamic change in 2-hydroxybutyrate in response to a metabolic challenge is a more discriminating marker than a single fasting measurement [171,172,173]. Breath acetone is a volatile ketone body generated from acetoacetate and serves as a noninvasive, real-time marker of ketone metabolism. With appropriate standardization of environmental conditions and instrumentation, joint analysis of postprandial suppression and post-exercise recovery curves for breath acetone, together with fluorodeoxyglucose positron emission tomography (FDG-PET) or plasma ketone measurements, allows quantitative assessment of the dynamics of ketone suppression and subsequent reactivation of ketone utilization [174,175,176]. This non-invasive monitoring bridges the gap between systemic metabolism and myocardial fuel preference. Furthermore, stress-responsive cytokines known as mitokines, specifically Growth differentiation Factor-15 (GDF-15) and Fibroblast growth factor-21 (FGF-21), serve as systemic reporters of myocardial bioenergetic failure. These proteins are secreted by the heart in response to mitochondrial stress and integrate metabolic dysfunction with systemic inflammation, correlating strongly with adverse outcomes in HFpEF [177,178]. Within the endothelial-microvascular axis, circulating levels of asymmetric and symmetric dimethylarginine, which inhibit the arginine-NO pathway, together with arginine/citrulline ratios, cGMP, endothelin-1, angiopoietin-2, von Willebrand factor, and soluble intracellular adhesion molecule 1 (ICAM-1)/vascular cell adhesion molecule 1 (VCAM-1), collectively constitute the biomarker panel for coronary microvascular dysfunction and inflammatory endothelial activation [179,180,181,182]. These abnormalities are particularly frequent in HFpEF. Circulating biomarker levels can be substantially distorted by renal function, concomitant therapies including angiotensin-converting enzyme inhibitors, angiotensin receptor-neprilysin inhibitors and phosphodiesterase inhibitors, as well as systemic inflammation [183,184,185,186]. Interpretation is strongest when metabolic markers are co-interpreted with congestion, fibrosis and renal/hemodynamic indices, emphasizing concordant multi-axis patterns rather than single markers [187,188]. In the clinical evaluation of metabolic flexibility, assessment begins with a static fasting baseline panel that includes ketone bodies, lactate/pyruvate, LCACs, free fatty acids and insulin/homeostatic model assessment of insulin resistance (HOMA-IR), with optional inclusion of BCAAs/BCKAs, C3, C5 and 2-hydroxybutyrate. Repeat sampling 60–120 min after a mixed meal or 10–20 min of light-intensity exercise then provides suppression and recovery curves, preferably with concurrent monitoring of exhaled acetone to characterize the temporal dynamics of metabolic flexibility. Subsequently, directional trends in endothelial–microvascular markers (e.g., cGMP, endothelin-1, and angiopoietin-2) should be co-interpreted with the core panel comprising natriuretic peptides, Cys-C, uACR, and troponin. This integrated approach enables the structural characterization of axis-specific discordances. Ultimately, it helps delineate the interplay among metabolic, hemodynamic, renal, and endothelial axes at the individual patient level [167,168,171,189,190,191,192].

### 4.3. Omics, Extracellular Matrix and Imaging-Derived Biomarkers

Within the fibrosis and remodeling axis, the balance between matrix synthesis and degradation is indexed by collagen propeptides PICP, PINP and PIIINP, the CITP-to-MMP-1 ratio, and extracellular matrix (ECM)–associated proteins such as periostin, osteopontin and tenascin-C [193,194,195,196,197]. Progressive elevations in these biomarker profiles generally coincide with increasing myocardial stiffness, yet their interpretation is often confounded by hepatic or renal dysfunction [183,195,198,199,200,201]. In contrast, circulating miRNAs offer a high-resolution molecular landscape of metabolic remodeling. Specific miRNAs have been directly linked to the regulation of substrate metabolism. For instance, MiR-208a regulates the Mediator complex subunit 13 (MED13) to control whole-body energy expenditure and thyroid hormone signaling, while miR-21 modulates fatty acid oxidation via the peroxisome proliferator-activated receptor-α (PPAR-α) pathway [202,203]. Similarly, miR-126 reflects endothelial integrity, linking microvascular density to metabolic substrate delivery [204,205]. Proteomic and metabolomic signatures provide complementary insights [206,207,208,209]. BCAA-related signatures and short-chain acylcarnitine patterns (including C3 and C5) are particularly valuable for precise stratification of sub-phenotypes characterized by insulin resistance, lipotoxicity and mitochondrial oxidative dysfunction. However, substantial barriers impede their transition from bench to bedside. Most omics-derived signatures have been identified in small, homogeneous cohorts and lack validation in diverse populations with complex comorbidities. Furthermore, the lack of standardized analytical pipelines and high costs currently restrict these high-dimensional biomarkers to research tools rather than routine diagnostic instruments [168,210,211,212]. In imaging-based assessment, the biomarker is defined by the quantitative parameter rather than by the imaging modality itself. Myocardial PCr/ATP ratio measured by ^31^P-MRS provides an estimate of myocardial energetic reserve, myocardial triglyceride content quantified by ^1^H-MRS reflects lipotoxic accumulation, and the monoexponential clearance rate k_mono_ derived from ^11^C-acetate PET represents global oxidative flux and tricarboxylic acid cycle turnover [213,214,215,216,217]. ^18^F-FDG is used to assess myocardial glucose utilization, although interpretation requires caution in the presence of concomitant inflammation, whereas ^15^O-water permits quantitative evaluation of perfusion and coronary microvascular function [218,219,220,221]. Nevertheless, cost, accessibility, standardization, radiation exposure, and the contextual interpretation of imaging data remain substantial practical constraints. This underscores that while imaging provides deep physiological insight, its scalability for routine metabolic monitoring remains limited compared to circulating biomarkers. Clinical utility is maximized by applying these advanced biomarkers as phenotype-tailored extensions to a core biomarker panel.

### 4.4. Practical Clinical Implications

**Figure 2:**
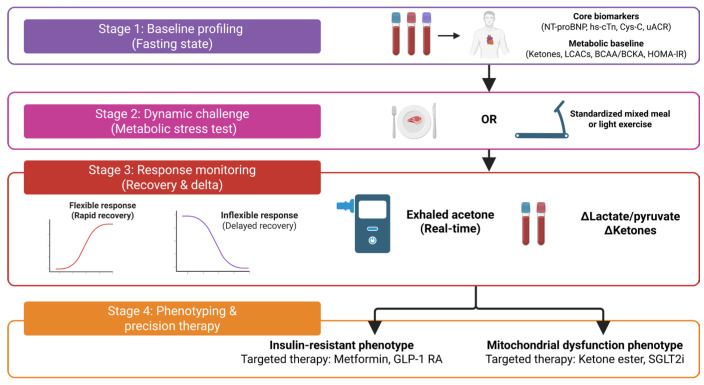
Integrated framework for the dynamic assessment of cardiac metabolic flexibility and precision phenotyping. This algorithm proposes a paradigm shift from static biomarker reliance to dynamic functional profiling. Stage 1 (Baseline Profiling): Establishes the patient’s metabolic baseline using a core panel of hemodynamic, renal, and static metabolic biomarkers including ketone bodies and long-chain acylcarnitines (LCACs) in the fasting state. Stage 2 (Dynamic Challenge): Introduces a standardized metabolic perturbation—either a mixed-meal tolerance test or a light exercise challenge—to stress the metabolic flexibility of the myocardium. Stage 3 (Response Monitoring): Quantifies the recovery kinetics of metabolic metabolites. A “Flexible Response” is defined by the rapid suppression and subsequent recovery of markers such as real-time exhaled acetone and the change in lactate-to-pyruvate ratio, whereas an “Inflexible Response” exhibits blunted or delayed kinetics. Stage 4 (Precision Therapy): Utilizes these dynamic signatures to stratify patients into specific metabolic endotypes, ranging from insulin-resistant phenotypes to mitochondrial-deficient phenotypes, guiding the selection of mechanism-based interventions such as glucagon-like peptide-1 receptor agonists (GLP-1 RAs) or sodium-glucose cotransporter 2 (SGLT2) inhibitors to optimize energetic efficiency. (Figure created in Biorender. Hyeong Rok Yun. (2025) https://app.biorender.com/illustrations/canvas-beta/6931987684378dd32321cd45, accessed on 13 January 2026).

To translate these concepts into practice, we delineate a four-stage integrated framework as illustrated in Figure 2. The process begins with establishing a metabolic baseline (Stage 1) using static markers such as fasting ketone bodies and LCACs. This is followed by a dynamic challenge (Stage 2), utilizing a mixed-meal or exercise test to perturb metabolic homeostasis. The core assessment lies in response monitoring (Stage 3), where the kinetics of marker suppression and recovery distinguish a “flexible” from an “inflexible” phenotype. Finally, these dynamic signatures guide precision therapy (Stage 4), allowing clinicians to select mechanism-based interventions such as GLP-1 RAs for insulin-resistant phenotypes or SGLT2 inhibitors for hemodynamic congestion based on the specific metabolic defect identified.

The clinical implementation of this framework facilitates a shift from generic management to phenotype-specific targeting. In routine clinical practice, differentiating the obese-metabolic phenotype (elevated HOMA-IR, leptin) from the hemodynamic-congestive phenotype (elevated BNP, CA125) is crucial. Consistent with STEP-HFpEF, targeting metabolic drivers with GLP-1 receptor agonists can benefit an obesity-related phenotype, whereas a congestion-dominant profile may prioritize SGLT2 inhibition and diuretics [122,222]. Furthermore, this biomarker-guided approach is critical for the design of future clinical trials. Moving beyond all-comers enrollment to biomarker-enriched strategies, where patients are selected based on specific defects such as blunted ketone suppression or acylcarnitine abnormalities, will be essential to successfully validate novel mitochondrial modulators [12].

Consistent with the 2022 AHA/ACC/HFSA guidelines, the foundational clinical panel must include BNP/NT-proBNP and high-sensitivity troponin to define structural status [84,223,224]. However, for metabolic phenotyping, these must be supplemented by Cys-C (±eGFR) and uACR [225]. In the context of therapy, special attention is required for patients receiving SGLT2 inhibitors. In these patients, mild elevations in circulating ketones should be interpreted with caution. Recent evidence suggests this often reflects a pharmacologically induced adaptive shift toward more efficient fuel utilization rather than metabolic decompensation alone [226,227]. Crucially, the diagnostic power of metabolic markers is amplified by calculating “delta values”, the quantitative change pre- and post-challenge, rather than relying on absolute cutoffs. However, a critical distinction must be made regarding biomarker maturity. Unlike natriuretic peptides and troponin, which benefit from robust, standardized automated assays, many emerging metabolic and omics-derived biomarkers currently lack analytical harmonization. Inter-platform variability (e.g., between different mass spectrometry assays) and the absence of large-scale validation cohorts preclude the immediate use of universal diagnostic cut-offs [228,229]. Therefore, until standardized reference ranges are established, the clinical utility of these novel markers lies primarily in monitoring intra-individual trends rather than single-point diagnosis. The overarching interpretive principle prioritizes multi-axis combinations and longitudinal trajectories over isolated absolute values, aiming for precision paneling that accounts for phenotype, sex and comorbidities.

## 5. Conclusions

Metabolic flexibility is a central determinant of myocardial resilience. Loss of metabolic flexibility, driven by diverse cellular and systemic dysfunction, acts as a unifying pathophysiological mechanism in both HFrEF and HFpEF. The clinical assessment of heart failure must evolve from reliance on isolated static metrics toward a dynamic, multi-axis framework. By integrating core hemodynamic markers with granular indices of metabolic flux, redox status, and tissue remodeling, clinicians can construct a high-resolution phenotype of the individual patient. Ultimately, bridging the translational gap between metabolic mechanisms and clinical practice requires the adoption of this structured biomarker hierarchy, facilitating a shift from generic management to precision metabolic interventions.

## Figures and Tables

**Table 1:** Key regulatory mechanisms and signaling pathways governing cardiac substrate switching.

Substrate	Key Regulators & Transports	Signaling Pathways & Mechanisms	Physiological Role in Metabolic Flexibility
Fatty acids (FA)	CD36, CPT-1	**PPAR-α/PGC-1-α axis**: upregulates FA uptake and β-oxidation enzymes. **ACC2/Malonyl-CoA**: inhibits CPT-1, regulating FA entry into mitochondria.	Primary fuel source (60–90%); utilization increases during fasting or insulin resistance but suppresses glucose oxidation (Randle Cycle).
Glucose	GLUT1, GLUT4	**PDH complex**: gatekeeper of glucose oxidation; inhibited by PDK4 and high Acetyl-CoA. Insulin/PI3K/AKT: promotes GLUT4 translocation and glucose uptake.	Essential for responding to increased workload and ischemia; strictly suppressed by FA oxidation under normal conditions.
Ketone body	MCT1, BDH1, SCOT	**Mass action effect**: uptake is proportional to circulating concentration. **NADH/NAD^+^ redox state**: ketone oxidation increases mitochondrial Acetyl-CoA, inhibiting PDH and sparing glucose.	“Thrifty fuel” during stress or starvation; improves hydraulic efficiency and reduces oxidative stress compared to FA.
Branched-chain amino acids (BCAA)	BCKDH	**mTORC1 pathway**: inhibits autophagic turnover and promotes protein synthesis. **KLF15**: Transcriptional regulator of BCAA catabolism.	Minor fuel contribution; accumulation of BCKAs (due to defective BCKDH) triggers mTOR-mediated insulin resistance and oxidative stress.

## Data Availability

No new data were created or analyzed in this study. Data sharing is not applicable to this article.

## Abbreviations

ACC	Acetyl-CoA carboxylase 2
AF	Atrial fibrillation
ATP	Adenosine triphosphate
BCAA(s)	Branched-chain amino acid(s)
BCKA(s)	Branched-chain α-keto acid(s)
BCKDH	Branched-chain α-keto acid dehydrogenase complex
BDH1	β-Hydroxybutyrate dehydrogenase 1
BNP	B-type natriuretic peptide
BHB	β-Hydroxybutyrate
CA125	Carbohydrate antigen 125
CACT	Carnitine–acylcarnitine translocase
CKD	Chronic kidney disease
CITP	Carboxy-terminal telopeptide of type I collagen
CPT1/2	Carnitine palmitoyltransferase-1/2
Cys-C	Cystatin C
EAT	Epicardial adipose tissue
ECM	Extracellular matrix
FDG	Fluorodeoxyglucose
GLP-1 RA	Glucagon-like peptide-1 receptor agonist
GLUT1/4	Glucose transporter 1/4
HFrEF	Heart failure with reduced ejection fraction
HFpEF	Heart failure with preserved ejection fraction
HOMA-IR	Homeostatic model assessment of insulin resistance
ICAM-1	Intercellular adhesion molecule-1
IDH	Isocitrate dehydrogenase
LCAC(s)	Long-chain acylcarnitine(s)
LCFA(s)	Long-chain fatty acid(s)
LVEF	Left ventricular ejection fraction
MCT1/4	Monocarboxylate transporter-1/4
MR-proADM	Mid-regional pro-adrenomedullin
mETC	Mitochondrial electron transport chain
mTORC1	Mechanistic target of rapamycin complex 1
NO	Nitric oxide
NT-proBNP	N-terminal pro-B-type natriuretic peptide
PCr	Phosphocreatine
PCr/ATP	Phosphocreatine-to-ATP ratio
PDH	Pyruvate dehydrogenase
PDK	Pyruvate dehydrogenase kinase
PGC-1α	Peroxisome proliferator–activated receptor-γ coactivator-1α
PKG	Protein kinase G
RAAS	Renin–angiotensin–aldosterone system
ROS	Reactive oxygen species
SCOT	Succinyl-CoA:3-ketoacid CoA transferase
SERCA2a	Sarcoplasmic/endoplasmic reticulum Ca^2+^-ATPase 2a
SGLT2	Sodium–glucose cotransporter-2
SIRT3/5	Sirtuin-3/5
TCA	Tricarboxylic acid
TFAM	Mitochondrial transcription factor A
TMAO	Trimethylamine N-oxide
uACR	Urinary albumin-to-creatinine ratio
VCAM-1	Vascular cell adhesion molecule-1
VLDL	Very-low-density lipoprotein
